# Sports participation and psychosocial health: a longitudinal observational study in children

**DOI:** 10.1186/s12889-018-5624-1

**Published:** 2018-06-07

**Authors:** Janet Moeijes, Jooske T. van Busschbach, Ruud J. Bosscher, Jos W. R. Twisk

**Affiliations:** 1 Department of Human Movement and Education, Windesheim University of Applied Sciences, Campus 2-6, Zwolle, 8017 CA The Netherlands; 2 University Medical Center Groningen, University Center for Psychiatry, PO Box 30001, 9700 RB Groningen, The Netherlands; 3 Department of Public and Occupational Health, Amsterdam Public Health research institute (VUmc), Van der Boechorststraat 7, Amsterdam, 1081 BT The Netherlands; 4 Department of Epidemiology and Biostatistics, VU Medical Center Amsterdam, Van der Boechorststraat 7, 1081BT Amsterdam, The Netherlands

**Keywords:** Children, Different characteristics of sports participation, Internalizing problems, Externalizing problems, Prosocial behaviour, Longitudinal associations

## Abstract

**Background:**

It is well known that sports participation is positively associated with psychosocial health in children, but details about this association over time are lacking. This study aimed to explore longitudinal associations between several characteristics of sports participation and three aspects of psychosocial health (internalizing problems, externalizing problems and prosocial behaviour) in Dutch children aged 10–12 years.

**Methods:**

Data from 695 fourth-grade primary school children were included at baseline; 10–13 months later, 487 children (response rate 70.1%) were retained. At both time points, children reported on their sports participation (Move and Sports Monitor Questionnaire – Youth Aged 8–12 Years) and psychosocial health (Strength and Difficulties Questionnaire). Longitudinal associations between several characteristics of sports participation and the three aspects of psychosocial health were analysed using linear mixed models, both clustering the repeated measures within children and clustering the children within schools. In addition to crude analyses, analyses were performed adjusting for sex, age, BMI, household composition, SES and frequency of sports participation.

**Results:**

Membership in a sports club, moderate or high frequency of sports participation, performing team sports, performing outdoor sports, performing contact sports and involvement in competition were longitudinally associated with fewer internalizing problems. The longitudinal association of higher frequency of sports participation with fewer internalizing problems was stronger as a child’s BMI increased. The association of performing team sports with fewer internalizing problems was only observed for boys. Membership in a sports club and moderate or high frequency of sports participation were also longitudinally associated with better prosocial behaviour. These associations with prosocial behaviour were stronger for girls. None of the characteristics of sports participation examined were longitudinally associated with externalizing problems.

**Conclusions:**

This study shows that from a longitudinal perspective, fewer internalizing problems and better prosocial behaviour were seen in children who were active in sports. Fewer internalizing problems were also associated with the kind of sports participation; for example, with performing outdoor sports. No associations were found for externalizing problems. Future research should preferably take the form of an intervention to investigate whether the observed statistical associations are of a causal nature.

## Background

A substantial number of children worldwide suffer from psychosocial health problems [1], which often continue into adulthood [2–4]. The concept of a child’s psychosocial health can be defined, on the one hand, as the absence or lower levels of negative traits, such as internalizing problems (i.e., emotional problems and peer problems) and externalizing problems (i.e., conduct disorders and hyperactivity-inattention), and, on the other hand, as the presence, or higher levels, of positive traits such as appropriate social skills [5–7]. Fewer internalizing problems, fewer externalizing problems and better prosocial behaviour reflect better psychosocial health [7]. A child’s psychosocial health is not determined in isolation, but is influenced by various social, economic and physical factors [8].

One of these influential factors is sports participation. Studies in adults, adolescents and children show that sports participation has beneficial psychosocial health outcomes [9–12], but details about the relationship between sports participation and psychosocial health are often lacking [13]. Most studies in this field focus primarily on internalizing problems, while the relationship between sports participation and externalizing problems or prosocial behaviour has not often been taken into account. Furthermore, the studies available on the relationship between a child’s sports participation and psychosocial health cover only a limited number of characteristics of sports participation. They mainly focus on whether a child participates in sports activities on a regular basis or not [14–16] or the distinction between individual and team sports [15]. Moreover, there are only a few studies with a longitudinal design [16–19]. Compared to a cross-sectional study, a longitudinal study has the advantage of relating the individual development of a certain outcome variable over time to the individual development of, or changes in, other variables [20].

The purpose of this study was to explore whether and how various characteristics of sports participation were longitudinally associated with internalizing problems, externalizing problems and prosocial behaviour in Dutch children aged 10–12 years.

## Methods

### Study design and participants

Data were obtained within a national-representative study, examining sports participation and psychosocial health in fourth and fifth-grade primary school children in the Netherlands. The children in the sample under study were geographically fairly well spread across the Netherlands and came from both rural and urban areas. Furthermore, our sample was comparable with the general Dutch population of primary school children for body mass index (BMI), neighbourhood socioeconomic status (SES) and sports participation (in terms of membership in a sports club).

The study included a cross-sectional component and a longitudinal component. Data were collected from November 2011 to June 2014.

Primary schools were first contacted by telephone and email. Interested schools were sent an email with detailed information and were subsequently approached with additional details and to make an appointment for a visit. If permission was given, the school sent written information about the aim, nature and practical process of the research to the parents and guardians of the children. Written informed consent from a parent or guardian was a precondition for participation in the research.

At the initial meeting, participating children were provided with a booklet containing questionnaires to be completed in the classroom. These completed questionnaires were collected 1 week later. Subsequently, anthropometric data were obtained during school time.

For the longitudinal component of the study, 998 children were invited to participate, with 752 (75.2%) responding positively. Due to missing data, ultimately 695 fourth-grade primary school children (69.6%; 334 boys, 361 girls; 29 primary schools) were included in the sample for the first measurement. After a period of 10 to 13 months, 508 children (73.1%) were retested (now in fifth grade). Due to missing data, 21 retested children (3.0%) were excluded from the analyses, resulting in a study sample of 487 children (response rate 70.1%; see also Statistical analyses).

The study was conducted in accordance with the guidelines proposed by the World Medical Association Declaration of Helsinki and was approved by the Medical Ethical Committee at VU University Medical Center Amsterdam (Twisk: 12/151). All parents and guardians of children in the sample provided a written informed consent.

### Measures

#### Psychosocial health

Psychosocial health was measured by the self-report version of the Strengths and Difficulties Questionnaire (SDQ), which assesses psychosocial health [21]. Apart from its clinical application, the SDQ has evolved into a screening method in research settings [22, 23] determining strengths and difficulties of children [24]. Due to its brevity and simplicity, the SDQ has become one of the most widely used screening instruments regarding children’s psychosocial health [25, 26]. The inclusion of both strengths and difficulties in the SDQ makes the questionnaire suitable for studies in psychosocially healthy children [27].

The five original subscales of the SDQ measured emotional symptoms, peer problems, conduct problems, hyperactivity-inattention and prosocial behaviour, subsequently organized into three broader scales for healthy subjects [7]. Emotional problems and peer problems form the ‘internalizing problems scale’. Conduct disorders and hyperactivity-inattention form the ‘externalizing problems subscale’ and the original prosocial behaviour subscale remained unchanged. The 25 items in the questionnaire are formulated as statements with response categories ranging from ‘not true’, ‘somewhat true’ to ‘certainly true’.

We followed the guidelines in the official manual of the SDQ [25] on subscale calculation and missing data. The internal consistency of each of the scales – the internalizing problems subscale, the externalizing problems subscale and the prosocial behaviour subscale – is reflected in Cronbach alphas of 0.66, 0.76 and 0.66 respectively [7]. The test-retest reliability of the Dutch self-report version of the SDQ subscales, expressed in an intraclass correlation coefficient (ICC), is 0.76 or higher, except for the prosocial behaviour subscale, which has an ICC of 0.59 [28].

#### Sports participation

The study focused on sports activities in sports clubs, the main environment in which Dutch children participate in sports [29]. A child’s participation in sports club activities was measured using two items from the self-report Move and Sports Monitor Questionnaire (MSMQ) – Youth Aged 8–12 Years [30]: membership in a sports club and frequency of training and matches per week. We added a third question about the type of sport(s) in which a child participated, as a means of distinguishing between different kinds of sports.

Sports were defined as team sports when practised by two or more people who cooperate to attain an optimal result and perceive each other as allies, while individual sports were defined as those in which the child is active as a single player and perceives other participants as opponents. In addition, we distinguished between indoor versus outdoor sports and between contact sports such as football and judo and non-contact sports such as volleyball and swimming. The final characteristic of sports participation concerned whether a child was involved in competition or not. The validation of the questions about sports participation was described in Moeijes et al. [31].

### Covariates

The covariates of age (continuous), sex (dichotomous), body mass index (BMI; continuous), household composition (dichotomous) and neighbourhood socioeconomic status (SES; continuous) were used in all analyses. Frequency of sports participation (continuous) was used as a covariate in the analyses of the associations between performing different kinds of sports (i.e. individual versus team sports, indoor versus outdoor sports, contact versus non-contact sports and involvement in competition or not) and psychosocial health. This was done to avoid the risk of finding spurious associations or overlooking important associations.

Parents or guardians reported the child’s date of birth and sex. Height and weight were measured by the researchers who visited the schools. Height was measured by the Seca 201 or 203 system (Basel, Switzerland) and weight by the Seca Sensa 804 (Basel, Switzerland) or the Tanita BC 601 digital scale (Tokyo, Japan). BMI was calculated as weight divided by height squared (kg/m^2^) [32]. Household composition was measured as living in a two-parent family or in another type of household, for example a one-parent family [33]. The neighbourhood SES of the child’s parents or guardians was based on postcodes and the status score per postcode derived from the Dutch Social and Cultural Planning Office in 2010 [34].

### Statistical analyses

Children who failed to answer one or more questions concerning the sports participation questionnaire MSMQ, BMI, SES or household composition were excluded from the analyses. Children who had missing data concerning the SDQ were excluded according the procedure outlined in the SDQ manual, which meant that for each of the original five subscales it was permitted to have one or two items missing. A greater number of missing data resulted in the exclusion of the child from the analyses.

All data were analysed using IBM SPSS (Version 24, IBM, New York, United States). Data on the outcome variables (internalizing problems, externalizing problems and prosocial behaviour), independent variables (membership in a sports club, frequency of sports participation classified into tertiles, individual versus team sports, indoor versus outdoor sports, contact versus non-contact sports and involvement in competition) and covariates (sex, age, BMI, SES, household composition and frequency of sports participation) for the children who did not participate in the second measurement were compared with the data for the children who participated in both measurements using independent t-tests or Pearson χ2-tests.

To control for the influence of prior testing, an additional sample of fifth graders was also tested (*N* = 450; 52.4% male, mean age = 12.0 years, SD = 0.5; 47.6% female, mean age = 12.0 years, SD = 0.5).

The longitudinal relationships between the characteristics of sports participation and psychosocial health (internalizing problems, externalizing problems and prosocial behaviour) were analysed using linear mixed models, taking into account the three-level structure of the data: repeated measures were clustered within children and children were clustered within the schools. Before analysis, normality of the distribution of the outcome variables was assessed by visual examination of histograms, which showed that internalizing problems subscale was skewed to the right. This subscale has therefore been log transformed.

In addition to crude analyses, analyses were performed adjusting for sex, age, BMI, household composition, SES and frequency of sports participation. Further analyses were performed to detect effect modifications, with interaction terms for all covariates analysed separately. For all analyses, a two-tailed significance level of *p* < 0.05 was considered statistically significant.

## Results

Of the 695 fourth-grade primary school children who filled in the questionnaires in the first test, 487 (70.1%) also fully completed the questionnaires in the retest (fifth grade) and were included in the study. The 208 children (29.9%) who were excluded did not participate in the retest because they were not interested, or were excluded due to missing data.

Children who only provided information for the first test measurement differed significantly from the children who were measured twice, with respect to age (11.1 versus 11.0 years, *p* = 0.002) and BMI (18.1 versus 17.6, *p* = 0.02) at the moment of the first test. No significant difference was found for sex, SES, household composition, membership in a sports club, frequency of sports participation and the outcome variables.

There was also no influence of prior testing observed, as the scores on the outcome variables did not differ significantly between children who were measured twice and the control group of children, who were only measured in the fifth grade (internalizing problems: *t* = 1.20, *p* = 0.23; externalizing problems: *t* = 0.63, *p* = 0.53; prosocial behaviour: *t* = 1.05, *p* = 0.29). Demographic characteristics of the entire sample are shown in Table 1.

**Table 1:** Demographic characteristics of the sample in the test (*N* = 695) and retest (*N* = 487)

		Fourth grade				Fifth grade			
		N	%	Mean	SD	N	%	Mean	SD
Sex	Boys	334	48.1			233	47.8		
	Girls	361	51.9			254	52.2		
Age				11.0	0.5			11.9	0.5
BMI				17.8	2.5			18.3	2.3
SES				0.25	0.91			0.30	0.90
Household composition	Two parent family	570	82.0			399	81.9		
	Others	125	18.0			88	18.1		
Membership sports club	No	89	12.8			63	12.9		
	Yes	606	87.2			424	87.1		
Frequency of sports participation				2.92	1.44			2.51	1.64
	Nonathletes	89	12.8			68	14.0		
	Low sports actives	229	32.9			130	26.7		
	Moderate sports actives	194	27.9			166	34.1		
	High sports actives	183	26.3			123	25.3		
Individual versus team sports	Nonathletes	89	12.8			68	14.0		
	Individual sports	161	23.2			108	22.2		
	Team sports	325	46.8			236	48.5		
	Individual as well as team sports	120	17.3			75	15.4		
Indoor versus outdoor sports	Nonathletes	89	12.8			68	14.0		
	Indoor sports	207	29.8			130	23.7		
	Outdoor sports	308	44.3			239	49.1		
	Indoor as well as outdoor sports	91	13.1			50	10.3		
Contact versus non-contact sports	Nonathletes	89	12.8			68	14.0		
	Non-contact sports	240	34.5			161	33.1		
	Contact sports	277	39.9			205	42.1		
	Non-contact as well as contact sports	89	12.8			53	10.9		
Involvement in competition or not	Nonathletes	89	12.8			68	14.0		
	Training activities	139	20.0			86	17.7		
	Competition	467	67.2			333	68.4		
Psychosocial health	internalizing problems			3.97	2.78			3.21	2.83
	normal	647	93.1			453	93.0		
	abnormal	48	6.9			34	7.0		
	externalizing problems			5.53	3.07			5.16	3.17
	normal	572	82.3			403	82.8		
	abnormal	123	17.7			84	17.2		
	prosocial behaviour			7.90	1.62			8.31	1.56
	normal	642	92.4			450	92.4		
	abnormal	53	7.6			37	7.6		

### Internalizing problems

Table 2 shows the results of the linear mixed model analyses relating characteristics of sports participation and the three indicators of psychosocial behaviour. In both crude and adjusted analyses, membership in a sports club and higher frequency of sports participation were associated with fewer internalizing problems. Furthermore, performing outdoor sports and performing contact sports were also associated with fewer internalizing problems. The same was found for involvement in competition, in comparison with only doing training activities.

**Table 2:** Longitudinal associations of examined characteristics of sports participation with internalizing problems (*N* = 487)

		Linear mix models					
		Raw analyses			Adjusted analyses^a^		
		B^b^	P	CI	B^b^	p	CI
Sports participation	Nonathletes	Reference			Reference		
	Membership sports club	−0.25	< 0.001***	− 0.37 to − 0.13	−0.25	< 0.001***	− 0.37 to − 0.13
Frequency of sports participation	High sports actives	Reference			Reference		
	Moderate sports actives	0.02	0.71	−0.09 - 0.13	0.04	0.42	−0.06 - 0.15
	Low sports actives	0.17	0.004**	0.05–0.28	0.13	0.02*	0.02–0.24
	Nonathletes	0.33	< 0.001***	0.18–0.47	0.32	< 0.001***	0.17–0.47
Individual versus team sports	Individual sports	Reference			Reference		
	Team sports	−0.10	0.09	− 0.21 - 0.02	− 0.08	0.20	− 0.20 - 0.04
	Individual as well as team sports	−0.01	0.87	−0.15 - 0.13	0.02	0.84	− 0.14 - 0.17
	Nonathletes	0.19	0.01**	0.05–0.34	0.14	0.10	−0.02 - 0.30
Indoor versus outdoor sports	Indoor sports	Reference			Reference		
	Outdoor sports	−0.28	< 0.001***	− 0.38 to − 0.17	−0.22	< 0.001***	− 0.34 to − 0.11
	Indoor as well as outdoor sports	− 0.10	0.20	− 0.25 - 0.05	− 0.08	0.30	− 0.24 - 0.08
	Nonathletes	0.10	0.16	−0.04 - 0.24	0.10	0.23	−0.06 - 0.25
Contact versus non-contact sports	Non-contact sports	Reference			Reference		
	Contact sports	−0.19	0.001***	− 0.30 to − 0.09	− 0.12	0.05*	−0.25 - 0.00
	Non-Contact as well as contact sports	−0.07	0.34	−0.22 - 0.08	− 0.05	0.53	− 0.20 - 0.10
	Nonathletes	0.16	0.02*	0.02–0.29	0.19	0.01**	0.05–0.32
Involvement in competition or not	Competition	Reference			Reference		
	Training activities	0.16	0.003**	0.05–0.27	0.09	0.15	−0.03 - 0.22
	Nonathletes	0.30	< 0.001***	0.17–0.43	0.29	0.01**	0.08–0.43

^a^Adjusted for sex, age, BMI, neighbourhood SES and household composition. For the variables individual versus team sports, indoor versus outdoor sports, contact versus non-contact sports, and involvement in competition, the analyses were also adjusted for frequency of sports participation

^b^Unstandardized regression coefficient

Italic values represent statistically significant results; *p ≤ .05; **p ≤ .01; ***p ≤ .001

BMI and sex were significant effect modifiers. Children with higher BMI showed a stronger longitudinal association between higher frequency of sports participation and fewer internalizing problems compared to children with lower BMI. In addition, a longitudinal association between performing team sports and fewer internalizing problems was found, but only for boys.

### Externalizing problems

Regarding externalizing problems, no significant longitudinal associations with different characteristics of sports participation (Table 3) were found. Moreover, no significant effect modifications were observed.

**Table 3:** Longitudinal associations of examined characteristics of sports participation with externalizing problems (*N* = 487)

		Linear mix models					
		Raw analyses			Adjusted analyses^a^		
		B^b^	P	CI	B^b^	p	CI
Sports participation	Nonathletes	Reference			Reference		
	Membership sports club	−0.22	0.44	−0.77 - 0.33	− 0.30	0.28	− 0.86 - 0.25
Frequency of sports participation	High sports actives	Reference			Reference		
	Moderate sports actives	−0.10	0.67	−0.57 - 0.37	− 0.09	0.72	− 0.56 - 0.39
	Low sports actives	−0.04	0.88	−0.54 - 0.47	−0.02	0.94	−0.53 - 0.49
	Nonathletes	0.17	0.62	−0.49 - 0.82	0.27	0.43	−0.39 - 0.93
Individual versus team sports	Individual sports	Reference			Reference		
	Team sports	0.03	0.91	−0.50 - 0.56	−0.04	0.87	−0.59 - 0.50
	Individual as well as team sports	0.10	0.75	−0.53 - 0.74	−0.10	0.77	−0.79 - 0.59
	Nonathletes	0.25	0.45	−0.39 - 0.89	0.48	0.20	−0.25- 1.21
Indoor versus outdoor sports	Indoor sports	Reference			Reference		
	Outdoor sports	−0.21	0.42	0.73–0.30	−0.34	0.23	−0.88 - 0.21
	Indoor as well as outdoor sports	0.19	0.59	−0.50 - 0.88	0.02	0.96	−0.72 - 0.75
	Nonathletes	0.14	0.66	−0.48 - 0.76	0.35	0.33	−0.35 – 1.05
Contact versus non-contact sports	Non-contact sports	Reference			Reference		
	Contact sports	0.16	0.53	−0.34 - 0.69	0.11	0.70	−0.46 - 0.69
	Non-Contact as well as contact sports	0.47	0.17	−0.21 - 1.15	0.31	0.42	−0.46 - 1.08
	Nonathletes	0.33	0.29	−0.27 - 0.93	0.48	0.17	−0.21 - 1.18
Involvement in competition or not	Competition	Reference			Reference		
	Training activities	0.21	0.41	−0.29 - 0.70	0.47	0.10	−0.10 - 1.04
	Nonathletes	0.29	0.32	−0.29 - 0.87	0.85	0.04*	0.06–1.64

^a^Adjusted for sex, age, BMI, neighbourhood SES and household composition. For the variables individual versus team sports, indoor versus outdoor sports, contact versus non-contact sports and involvement in competition, the analyses were also adjusted for frequency of sports participation

^b^Unstandardized regression coefficient

Italic value represents statistically significant result; *p < .05

### Prosocial behaviour

Membership in a sports club and moderate and high frequency of sports participation were longitudinally associated with better prosocial behaviour (Table 4). Additional analyses investigating effect modification demonstrated that the longitudinal relationship for membership and frequency was more pronounced for girls.

**Table 4:** Longitudinal associations of examined characteristics of sports participation with prosocial behaviour (*N* = 487)

		Linear mix models					
		Raw analyses			Adjusted analyses^a^		
		B^b^	P	CI	B^b^	p	CI
Sports participation	Nonathletes	Reference			Reference		
	Membership sports club	0.30	0.05*	0.00–0.60	0.40	0.01**	0.10–0.69
Frequency of sports participation	High sports actives	Reference			Reference		
	Moderate sports actives	−0.02	0.89	−0.28 - 0.24	0.01	0.93	−0.25 – 0.27
	Low sports actives	−0.16	0.25	−0.44 - 0.11	−0.24	0.08	−0.51 - 0.03
	Nonathletes	−0.37	0.04*	−0.73 to − 0.02	−0.51	0.004**	−0.86 to − 0.16
Individual versus team sports	Individual sports	Reference			Reference		
	Team sports	−0.13	0.34	−0.41 - 0.14	− 0.06	0.69	− 0.34 - 0.22
	Individual as well as team sports	−0.06	0.71	−0.40 - 0.27	−0.16	0.40	−0.52 - 0.21
	Nonathletes	−0.38	0.03*	−0.72 to − 0.03	−0.27	0.18	−0.66 - 0.12
Indoor versus outdoor sports	Indoor sports	Reference			Reference		
	Outdoor sports	0.08	0.57	−0.19 - 0.34	0.25	0.08	−0.02 - 0.53
	Indoor as well as outdoor sports	0.09	0.62	−0.27 - 0.46	0.03	0.89	−0.35 - 0.41
	Nonathletes	−0.25	0.15	−0.58 - 0.09	−0.15	0.44	−0.53 - 0.23
Contact versus non-contact sports	Non-contact sports	Reference			Reference		
	Contact sports	−0.16	0.22	− 0.42 - 0.10	0.08	0.60	−0.12 - 0.37
	Non-Contact as well as contact sports	−0.14	0.45	−0.50 - 0.22	−0.13	0.52	−0.53 - 0.27
	Nonathletes	−0.38	0.02*	−0.71 to − 0.05	−0.20	0.29	−0.60 - 0.17
Involvement in competition or not	Competition	Reference			Reference		
	Training activities	−0.10	0.45	− 0.37 - 0.16	−0.17	0.26	−0.49 - 0.04
	Nonathletes	−0.33	0.04*	−0.64 to − 0.02	−0.38	0.08	−0.48 - 0.13

^a^Adjusted for sex, age, BMI, neighbourhood SES and household composition. For the variables individual versus team sports, indoor versus outdoor sports, contact versus non-contact sports and involvement in competition, the analyses were also adjusted for frequency of sports participation

^b^Unstandardized regression coefficient

Italic values represent statistically significant results; *p ≤ .05; **p ≤ .01

## Discussion

### Main findings

This study investigated the longitudinal relationships between characteristics of sports participation and psychosocial health in children aged 10–12 years. Although the characteristics of sports participation examined appeared to be longitudinally associated with psychosocial health, there were clear differences for the three aspects of psychosocial health. The characteristics of sports participation examined were particularly associated with internalizing problems. There were substantially less associations found between characteristics of sport participation and prosocial behaviour and no associations at all with externalizing problems.

### Internalizing problems

The longitudinal associations observed between all characteristics of sports participation may be explained with the help of the intermediate constructs ‘sports self-concept’ and ‘self-esteem’. A higher sports self-concept is beneficial for a child’s self-esteem [19, 35, 36], which is reflected in fewer internalizing problems [15, 37].

The longitudinal associations observed between membership in a sports club, moderate or high frequency of sports participation and fewer internalizing problems are in line with the results of other studies [17, 19, 38–40]. An explanation of these longitudinal associations might be that engaging in sports activities enhances a child’s sports abilities [11, 41, 42] and consequently stimulates his or her sports self-concept [15, 42, 43].

The association observed between moderate or high frequency of sports participation and fewer internalizing problems was stronger for children with higher BMI. Compared to healthy-weight peers, children with overweight are likely to have a lower self-concept and thereby lower self-esteem, resulting in more internalizing problems [44, 45]. It is probable that their self-concept and self-esteem may only be improved by undertaking sports activities on a very regular basis.

Unlike girls, boys showed a longitudinal association between performing team sports and fewer internalizing problems. An explanation for this sex effect might be that success in sports activities is of greater importance for self-esteem in boys than in girls [36, 46]. As a consequence, boys might have a greater urge to perform well at sports in cooperation with their peers. Their joint efforts to achieve good sports results could lead to a stronger sense of belonging during sports activities [47], resulting in higher self-esteem.

One explanation for the longitudinal association observed between performing contact sports and fewer internalizing problems might be that contact sports take place in a context in which children explore the social borders of permitted physical contact. This might reduce the uncertainty of the child about how to behave towards peers [48] and thereby promote his or her self-esteem [49].

A recent study on the effects of participating in competition in adolescence [50] found the same positive effect on mental health as our study of children. Despite the fact that children do not always win such competitive sports matches, they do offer the opportunity to experience success and might therefore improve self-esteem [51].

Our finding that performing outdoor sports was longitudinally associated with fewer internalizing problems is also in accordance with some previous studies [52, 53]. These studies reported that being active outdoors is additionally beneficial, as the open air itself has a positive influence with respect to a reduction in depressive feelings, which constitute an essential element of internalizing problems.

### Externalizing problems

In line with the results of other recent studies [17, 18, 54, 55], we found no longitudinal association between characteristics of sports participation and externalizing problems. This might be explained with the help of the intermediate concept of ‘effortful control’. Externalizing problems can be considered a consequence of a lack of effortful control, which means an insufficient ‘ability to willfully or voluntarily inhibit, activate, or change (modulate) attention and behavior, as well as executive functioning tasks of planning, detecting errors, and integrating information relevant to selecting behavior’ [56]. Effortful control is a trait of an individual’s temperament, which mainly evolves between the ages of 2 and 7 [57]. The children in our sample, who were aged between 10 and 12 years, had probably already achieved such stability in their effortful control, such that this characteristic of their temperament was little influenced by participating in sports activities.

### Prosocial behaviour

Our finding that membership in a sports club and frequency of sports participation were longitudinally associated with better prosocial behaviour confirms what has been described in other studies [18, 54, 58]. The disciplinary effect of engagement in sports activities on the behaviour of children [18, 41] probably also applies more specifically to their prosocial behaviour [59, 60]. The repetition of rules, i.e. bringing specific rules of conduct to the attention of children regularly and giving them sufficient opportunity to put them into practice, is conducive to developing and sustaining adequate prosocial behaviour.

The more pronounced longitudinal associations for girls could be due to a difference between the sexes in how aggression and empathy are expressed during sports. Girls behave less aggressively than boys during sports activities because of both complex social and cultural expectations and the physical characteristics of boys and girls [61, 62]. Stanger et al. [61] found that the sports attitudes of men were more aggressive than those of women. Women have an attitude to sports which is characterized more by empathy, which tends to prevent them from acting aggressively towards other people [63] and is therefore conducive to the development of prosocial behaviour.

### Strengths and limitations

This study presents essential outcomes concerning longitudinal relationships between sports participation and psychosocial health in children aged 10–12 years. The external validity of the study outcomes was improved by gathering longitudinal data using a large-scale survey of children from a considerable number of regular primary schools throughout the Netherlands, covering a wide variety of household composition and socioeconomic status.

Unlike most other studies in this field, our study did not focus on one or more specific psychosocial disorder such as depression or behavioural problems, but on three general aspects of psychosocial health, namely the absence of internalizing and externalizing problems and the presence of prosocial behaviour.

A further strength of our study is that a substantial number of characteristics of sports participation were taken into account. Most studies on the relationship between sports participation and psychosocial health only focus on a limited number of characteristics of sports participation.

The final strength pertains to the advantage of longitudinal analyses compared with cross-sectional analyses. In the case of longitudinal analyses, the estimated relationships reflect both the within-subject relationships and the between-subject relationships. Furthermore, the observations within a child at different time points are highly correlated. This correlation is overlooked in cross-sectional analyses.

There are, however, also some limitations. Although the time spent in sports participation is probably more strongly associated with psychosocial health than membership in a sports club and frequency of sports participation [64], we did not take the duration of sports participation into account in our analyses. Using self-reported information from the children, rather than objectively measured data about physical activity, it was not possible to assess the time spent in sports participation in a valid way.

Another limitation is that the longitudinal observational design only provided the opportunity to determine statistical associations. Because no intervention was performed, no causal relationships could be established. Furthermore, the follow-up period for the longitudinal study was relatively short (i.e. 10–13 months) and the study only included children within the age range of 10 to 12 years. The outcomes cannot be generalized to other age groups.

Finally, only two confounders used in the statistical analyses related to a child’s social, cultural and economic circumstances, namely socioeconomic status and household composition. Because contextual factors might have an impact on the relationship between sports participation and psychosocial health, the study would have been strengthened by paying more attention to such factors.

### Future research

In this study, the numerous associations between characteristics of sports participation and internalizing problems were explained on the basis of the assumption that a higher sports self-concept is beneficial for a child’s self-esteem, which is reflected in fewer internalizing problems. Although there is some research available that supports this explanation, future research could explicitly pay attention to children’s sports self-concept and their self-esteem as potential explanatory mechanisms. Furthermore, future research should preferably take place in the form of an intervention in order to investigate whether the statistical associations found in our research are of a causal nature. Finally, future research should pay more attention to children’s social, cultural and economic circumstances. In this respect, the cultural and ethnic background of the children might also be considered.

## Conclusions

This study found that, from a longitudinal perspective, children who were active in sports exhibited fewer internalizing problems and better prosocial behaviour. Fewer internalizing problems were also associated with the kind of sports participation, for example with performing outdoor sports. No associations were found for externalizing problems. Future research should preferably take place in the form of an intervention to investigate whether the observed statistical associations are of a causal nature.

## Abbreviations

BMI: Body mass index
SDQ: Strengths and Difficulties Questionnaire
SES: Socioeconomic status
